# Promotion of awareness and utilization of youth friendly service through multi-sectoral cooperation mechanism in China

**DOI:** 10.1186/s12889-020-8313-9

**Published:** 2020-05-24

**Authors:** Ning Feng, Xi Jin, Jiuling Wu, Linhong Wang

**Affiliations:** 1 Center for Global Public Health, Chinese Center for Disease Control and Prevention, Room 211, 155 Changbai Road, Changping District, Beijing, People’s Republic of China; 2 National Center for Women and Children’s Health, Chinese Center for Disease Control and Prevention, 12 Dahuisi Road, Haidian District, Beijing, People’s Republic of China; 3 National Center for Non-communicable Disease Prevention and Control, Chinese Center for Disease Control and Prevention, 27 Nanwei Road, Xicheng District, Beijing, People’s Republic of China

**Keywords:** Youth friendly service, Awareness, Multi-sectoral cooperation, Reproductive health, Satisfactory degree

## Abstract

**Background:**

Youth friendly services (YFS) was established in pilot areas in China. This study aimed to explore the promoting level on the awareness and utilization of YFS after the implementing of a multi-sectoral cooperation mechanism (MSCM) supported by social network theory (SNT) among multiple sectors related to young people reproductive health (YRH) closely.

**Methods:**

A cross-sectional study with two separate self-administered questionnaire surveys was conducted before and after the implementing of a MSCM supported by SNT in both in-school and out-school unmarried young people aged 12–24 year-old in pilot areas in China. Both pre- and post- implementation surveys were conducted between December, 2008 and January, 2009, and between October, 2010 and January, 2011 respectively. The collected categorical data about the awareness on YFS in young people was described in percentage (%). χ ^2^ test was used to compare the differences between interventional and control areas, pre and after intervention, and changes in investigated areas after the intervention respectively. Binary logistic regression was used to analyze interventional effects after adjusting gender, in-school or out-school, and other factors. Significance level α was 0.05.

**Results:**

The percentages of young people in interventional areas who could receive YRH education including that about YFS in schools, working sites and communities increased (*OR* = 15.485, 6.166, 3.723; 95% *CI*: 2.939~4.715, 4.014~9.473, 11.421~20.994 respectively) statistically significantly (*P* < 0.05). The percentages of young people in interventional areas who “have heard of YFS clinic” and “know that YFS clinic has been established in local area” (*OR* = 9.325, 11.244; 95% *CI*: 7.433~11.699, 8.780~14.399 respectively), and knowledge rates on YFS manner and contents also increased (*OR* = 14.830, 8.676; 95% *CI*: 9.728~22.607, 5.175~14.548 respectively) statistically significantly (*P* < 0.05). The increments of knowledge rates on YFS price, time, hotline number, contents on contraception, pregnancy and sexual harass/violence were statistically significant (*P* < 0.05). The satisfaction degree on this service has also increased (*OR* = 6.394, 95% *CI*: 2.789~14.655) statistically significantly (*P* < 0.05).

**Conclusions:**

SNT is a helpful tool to facilitate the construction of an effective multi-sectoral cooperation mechanism to promote the awareness and satisfactory degree of YRH services.

## Background

Youth friendly service (YFS) is one of the most important public health strategies to promote reproductive health among young people in multiple aspects [1, 2]. It provides health-related information, health education and services regarding sexuality, contraception and sexually transmitted infections, including human immunodeficiency virus (HIV) infection. It also involves counseling young men about the importance of mutual respect towards decision of their female counterparts and their shared responsibility toward reproductive health (RH) [3, 4].

China started RH services for young people specially in 1990’s and integrated them into the existing public infrastructure of adolescent health services. The service was improved according to international YFS guidelines [1, 2]. YFS clinics were set up in public Maternal and Child Health (MCH) Care Centres at county level. There was no research focus on the promotion of the utilization of YFS clinics specially and systematically before this study. However, several investigations had shown the needs of YFS in young people in these areas [5–9]. Also, the initial reports stated that the clinics faced challenges in their utilization and there was an obvious gap between low utilization degree of YFS clinic services and high demand for the services. Since then, the model has been improved constantly to create a friendly environment toward young people [4, 10–14]. There were 1.39538 billion people living in China main land in 2018 [15] among them 16.86% were young people aged 10–24 year-old according to 0.824‰ sampling population survey conducted in 2017 [16]. Since young people are involved in various sectors across the country, a special multi-sectoral cooperation mechanism consisting of various sectors was established in pilot areas in 2008. In this study, we aimed to explore the level on promoting awareness and utilization of YFS after implementing multi-sectoral cooperation mechanism.

## Methods

### Study design

This was a cross-sectional study with two separate surveys conducted before and after the implementation of multi-sectoral cooperation mechanism in pilot areas in China; pre-implementation survey was conducted between December, 2008 and January, 2009, and post-implementation survey between October, 2010 and January, 2011 in the same areas.

As a first stage, YRH project counties in Guangdong, Chongqing and Guizhou provinces were selected as implementation areas while those in Hei Longjiang, Shandong and Jiangsu provinces were selected as controls by matching the Gross Domestic Products (GDPs) between these counties. They were selected because YFS clinics had been established there and they were proactive to participate in the intervention and/or investigation.

As second stage, one junior and one high middle schools were selected randomly. One class was selected from each grade of both junior and high middle schools (3 junior and 3 high school classes), based on the similarity of the number of girls and boys. Students were recruited by their seat order into the study. If the classroom size was less than 40, a class next to it was selected to recruit enough students.

For out-school young people enrollment, all unmarried young people aged 12–24 year-old from randomly selected communities or worksites in the investigated counties were included.

The investigated schools, communities and worksites for pre and post intervention were not the same.

### General setting

China is the second-largest country by land area in the world and the most populous country, with a population more than 1.3 billion. The country is divided into 22 provinces, 5 autonomous regions, and 4 municipalities (Beijing, Tianjin, Shanghai and Chongqing) and 3 special administrative regions. Geographically, all provincial divisions can be grouped into 6 regions, including North China, Northeast China, East China, South Central China, Southwest China and Northwest China. The provinces are divided into prefectures, districts/counties, communities/townships, neighborhood committees/villages (urban/rural).

### Multi-Sectoral cooperation mechanism

The research team developed “Action Plan on Promoting the Delivery and Utilization of YFS through Multi-Sectoral Cooperation Mechanism” which was shared with the control areas after one and a half years of implementation in pilot areas.

**Fig. 1:**
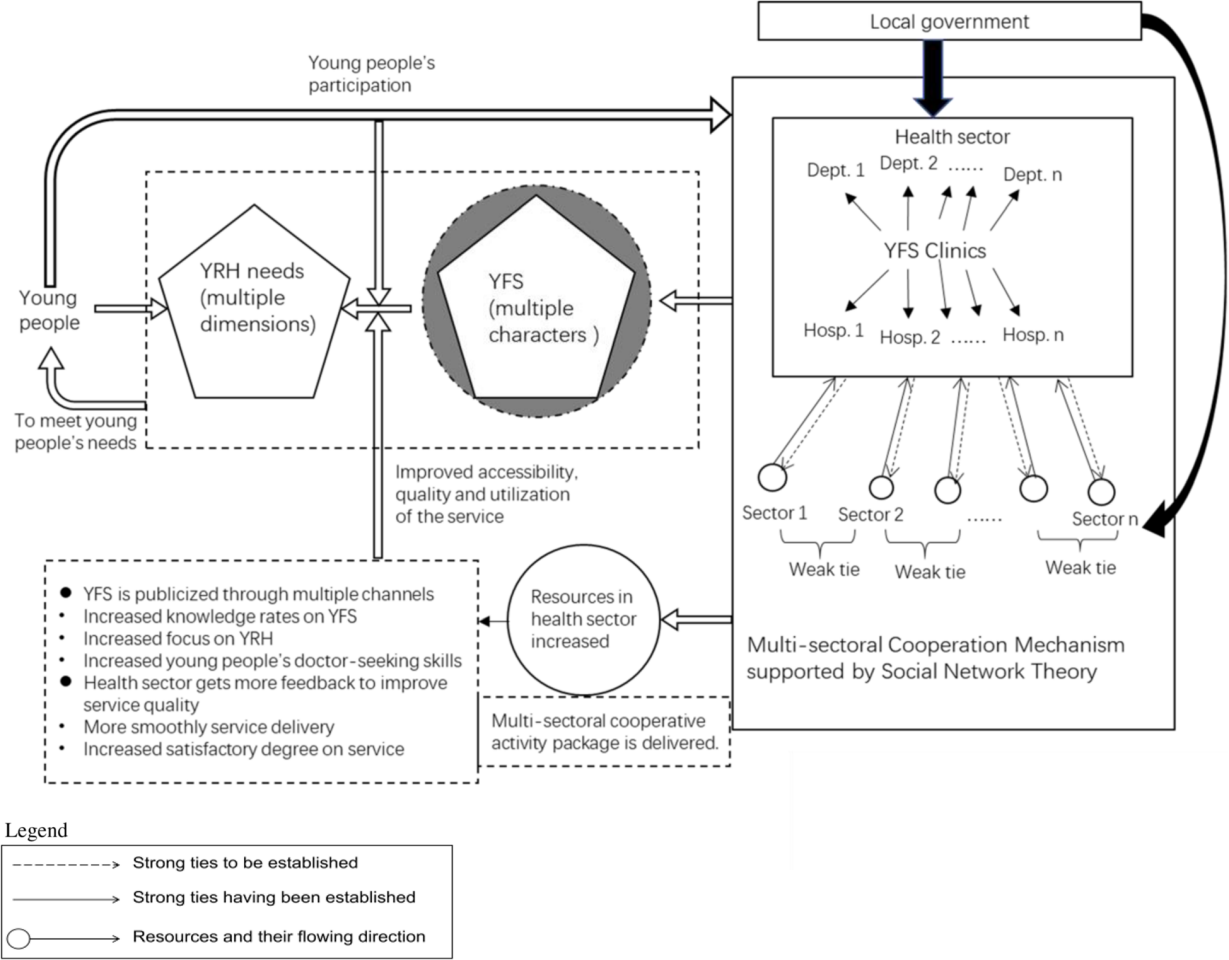
The Illustration of Promoting YFS through Multi-Sectoral Cooperation Mechanism

Social network theory (SNT) was used to support the construction of the multi-sectoral cooperation mechanism. Originally, within the health sector, strong ties existed between YFS clinic and other departments as well as related medical institutions. So the exchange of information and resources between them were systematic and rich but superfluous. Other sectors related to YRH closely such as those of education, Women’s Federation, the Communist Youth League, the media, community and China Youth Network (CYN) had less frequent interaction with the health sector but they could provide new resources and information to the health sector. Then ties between health sector and other related sectors were strengthened through activities to construct a multi-sectoral cooperation mechanism in order to push the resources and information flow from these sectors to the health sector. No cooperation task was designed between sectors outside health sector in YRH (Fig. 1).

**Fig. 2:**
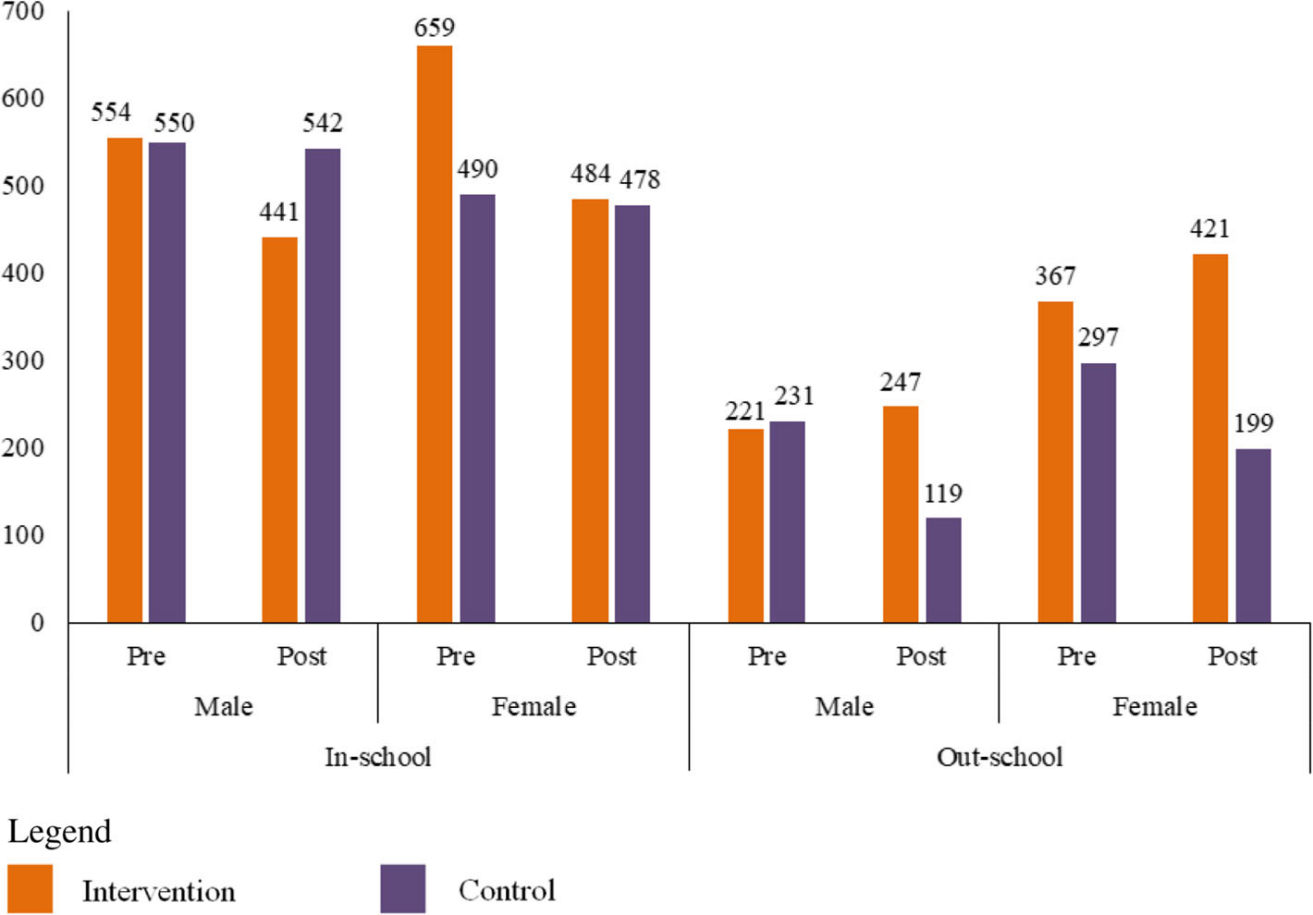
Numbers of Young People Participating the Questionnaire Survey Pre and Post Intervention (cases)

Based on the analysis of the resources in education, publicity, technique, facilities, materials and assistance etc. that could be provided by various sectors, responsibilities of each sector in the cooperation were clarified; the 4 dimensions (emotional strength, interaction frequency, intimacy and reciprocity exchange) to judge the strength of ties in SNT were corresponded to the conditions to establish multi-sectoral cooperation. Next, cooperative activities were designed (Table 1) [17].

**Table 1:** Multi-Sectoral Cooperation Activities Developed by Using SNT

SNT	MSCM	Actions	Activity Package
Emotional intensity	Common causes	Promote YFS utilization and YRH	NA
Interaction frequency	Stakeholder participation	Guarantee the frequency of coordination, communication and cooperative activities	For each year: Twice multi-sectoral coordination meetings · Once nation-level intensive training · At least 4 h’ curriculum on YRH and YFS · Twice lectures and Q&A activities · Regular publicity and lecture through various public media · Twice feedbacks from young people on the services · Once nation-level field technical supports · Twice local self-assessments
Reciprocal services	Confidence	Interactive supports in workforce, technique and funding	Multi-sectoral cooperative activities: · Regular coordination meetings to discuss and resolve problems on YRH and YFS · Improvements on YFS facilities, environment and quality to absorb young people · YRH education · Trainings in multi-sectors including service providers Young people’s feedback on YFS · Development and dissemination of various forms of IEC materials
Intimacy	Commitments/responsibilities	Strength of interactive supports	· Development and issuing of local administrative documents on MSCM to clarify the role of each sector and cooperative activities · Incorporation of each sector’s performance in the cooperation into Annual Review · Coordination by local government to ensure workforce, funding and materials needed · Technical support and assessment to identify and resolve problems timely

Notes: SNT is Social network theory, MSCM is Multi-sectoral cooperation mechanism

### Sample size calculation and sampling methods

Assuming the prevalence of the knowledge rate on YFS clinic in young people in the study setting to be 10% with the significance level set at 5%, and adjusting for a non-response of 10%, $n={\left(\frac{U_{\alpha }}{\delta}\right)}^2p\left(1-p\right)$ was used to calculate and the smallest needed sample size was 554. A stratified (schooling going versus out-of-school) multi-stage sampling strategy was used to achieve the required sample. For schooling going adolescents, the first stage sampling unit included counties or districts while the second sampling unit was school and the third stage students selected from each school. While, for out-school young people, the communities and enterprises that were willing to attend the survey and for which it was easy to organize young people were selected to recruit as many young people as possible. The reason for difference in recruitment method of in-school and out-school participants is because schooling young people were easier to organize than those of out-school ones. Before intervention, a total of 3369 young people were recruited from 12 schools (2 of each area), 9 communities and 15 enterprises. After intervention, 2931 young people were recruited from 12 schools (2 of each area), 5 communities and 19 enterprises (Fig. 2).

### Data collection

Investigator trainings were conducted before the surveys. Two fixed investigators along with two CYN members (one male and one female) conducted the surveys. The research team also conducted supervised technical support in implementation areas to ensure good quality implementation and services. The questionnaire was designed to understand the knowledge level on the existence of local YFS clinic, its services, and young people’s satisfaction level on it. The participants completed the self-administered questionnaires independently, and informed consent was taken before the administration of the survey. Although both questionnaire and group discussion were carried out for the whole study, this paper only reported the questionnaire part.

### Statistical analysis

The data collected was double-entered with validation using Epidata Entry version 3.1 and exported into Statistical Package for Social Science (SPSS) 11.5 for data analysis. The collected categorical data was described with percentage (%) and chi-square test was used to compare the differences between interventional and control areas. Logistic regression was used to determine the association between the intervention (exposure variables) and knowledge level as well as satisfactory degree (outcome variables). Unadjusted odd ratios with 95% Confidence Interval were calculated and those variables with *p*-value less than 0.2 were included in the multivariate model. Significance level α was set to be 0.05.

The statistically significant increasing of knowledge level on the existence of local YFS clinic, its service characters and contents, and young people’s satisfaction degree on it when compared with the control group were considered as interventional effects in this study.

## Results

Before intervention, a total of 3369 young people were recruited with mean ages of 15.8 and 20.8 years for in-school and out-school ones respectively. After intervention, a total of 2931 young people with mean ages of 15.9 and 21.2 years for in-school and out-school ones respectively. The difference for out-school young people was less than natural increment of age (1.5 years) (t = 4.684, *P* < 0.05).

### Young people’s knowledge rates on YFS clinics

**Fig. 3:**
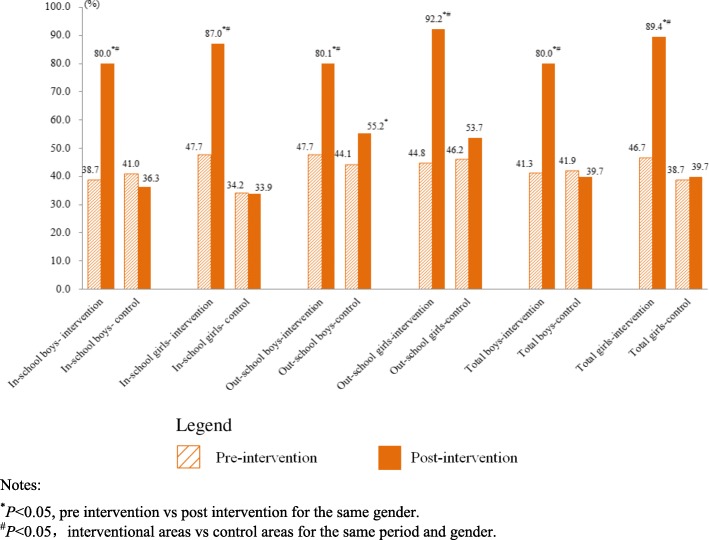
Percentages of the Young People Who “Have heard of YFS clinic” (%). Notes: ^*^P < 0.05, pre intervention vs post intervention for the same gender. Notes: ^#^*P* < 0.05, interventional areas vs control areas for the same period and gender

Before intervention, there was no statistically significant difference in the rates of “Have heard of YFS clinic” between interventional and control areas (*P* > 0.05). After intervention, this rate increased obviously in all 3 interventional areas and higher statistically significantly(χ^2^_in-school male_ = 386.244, χ^2^_in-school female_ = 274.255, χ^2^_out-scholl male_ = 23.448, χ^2^_out-school female_ = 167.768, *P* values < 0.05) than that of the 3 control areas in total for the same type and gender of young people (Table 2, Fig. 3).

**Table 2:**
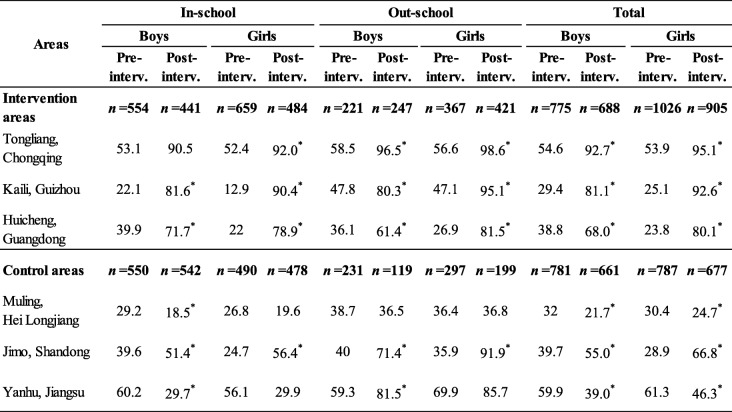
Percentages of the Young People Who “Have heard of YFS clinic” in investigated areas (%)

Notes

^*^P < 0.05, pre intervention vs post intervention for the same gender

^#^*P* < 0.05, interventional areas vs control areas for the same period and gender

**Fig. 4:**
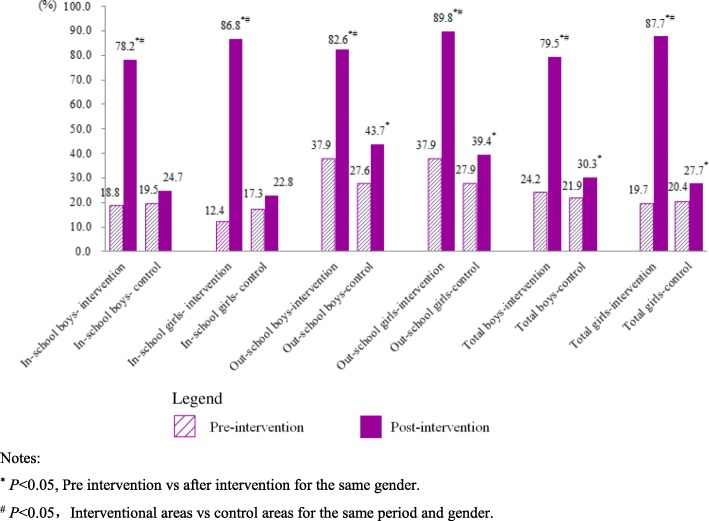
Percentages of the Young People Who “Know that YFS clinic has been established in local area” (%). Notes: ^*^P < 0.05, Pre intervention vs after intervention for the same gender. ^#^*P* < 0.05, Interventional areas vs control areas for the same period and gender

Before intervention, there was no statistically significant differences in the percentages of young people who “Know that YFS clinic has been established in local area” (*P* > 0.05). After intervention, the percentages in all 3 interventional areas increased obviously, and higher statistically significantly than those of young people with the same type (in-school or out-school) and gender respectively in control areas (χ^2^_in-school male_ = 355.920, χ^2^_in-school female_ = 3520.270, χ^2^_out-school male_ = 101.397, χ^2^_out-school female_ = 198.591, *P* < 0.05) (Table 3, Fig. 4).

**Table 3:**
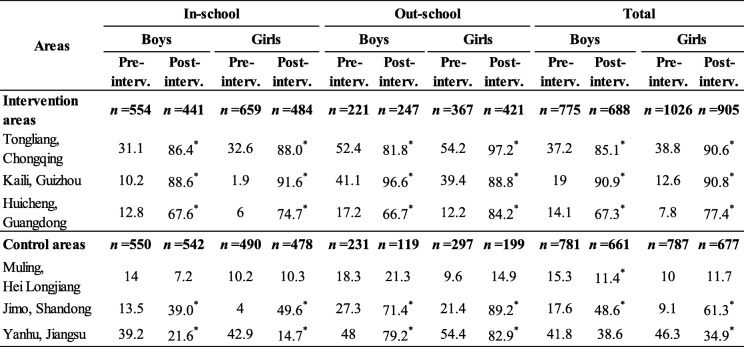
Percentages of the Young People Who “Know that YFS clinic has been established in local area” in investigated areas (%)

Notes

^*^P < 0.05, Pre intervention vs after intervention for the same gender

^#^*P* < 0.05, Interventional areas vs control areas for the same period and gender

### Knowledge rates on manner and content of YFS

**Fig. 5:**
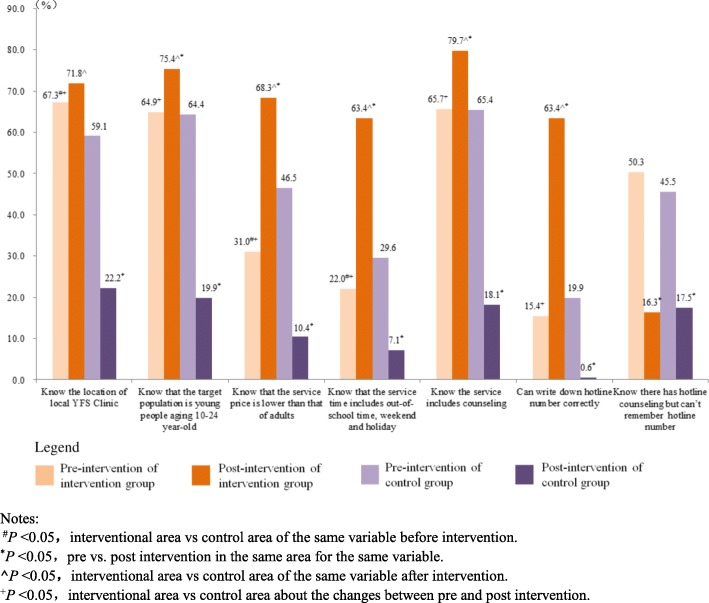
Knowledge Rates on Manner of YFS among Young People “Knowing that YFS clinic has been established in local area”(%). Notes: ^#^*P* < 0.05**,** interventional area vs control area of the same variable before intervention. ^*^P < 0.05, pre vs. post intervention in the same area for the same variable. **^***P* < 0.05**,** interventional area vs control area of the same variable after intervention. ^+^*P* < 0.05, interventional area vs control area about the changes between pre and post intervention

**Fig. 6:**
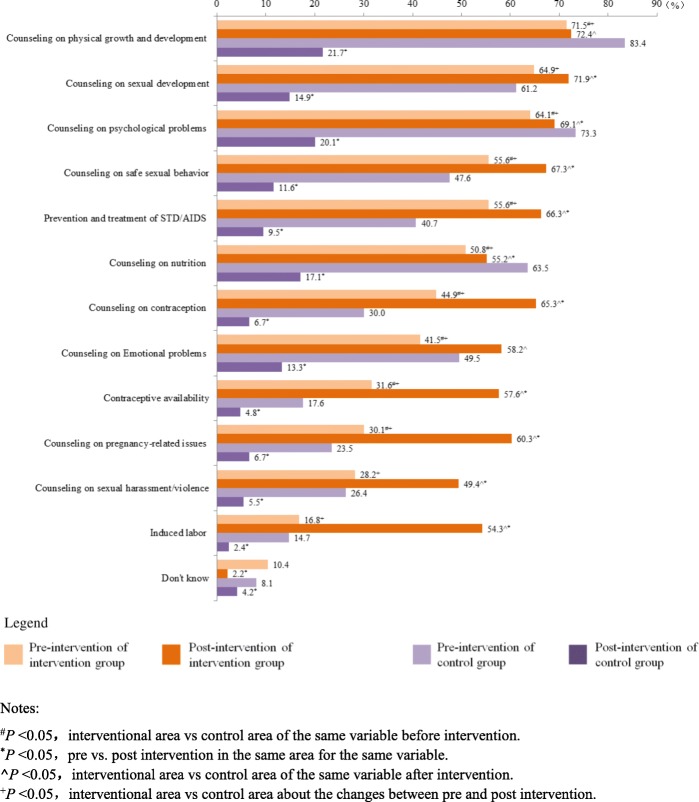
Knowledge Rates on Content of YFS among Young People “Knowing that YFS clinic has been established in local area”(%). Notes: ^#^*P* < 0.05**,** interventional area vs control area of the same variable before intervention. ^*^P < 0.05, pre vs. post intervention in the same area for the same variable. **^***P* < 0.05**,** interventional area vs control area of the same variable after intervention. ^+^*P* < 0.05, interventional area vs control area about the changes between pre and post intervention

After intervention period, among the young people who “Know that YFS clinic has been established in local area”, most of knowledge rates on YFS manner and content increased in interventional areas but decreased in control areas statistically significantly (*P* < 0.05) although 4 knowledge rates in control areas were higher than those in interventional with statistical significances (*P* < 0.05) before interventional period. The increment values in the knowledge rates on the prices, time, hotline number, contraception, pregnancy and sexual harass/violence of contents of YFS were larger comparatively (Fig. 5, Fig. 6).

### The relationship between the intervention and the improvement of knowledge and satisfaction degrees

**Fig. 7:**
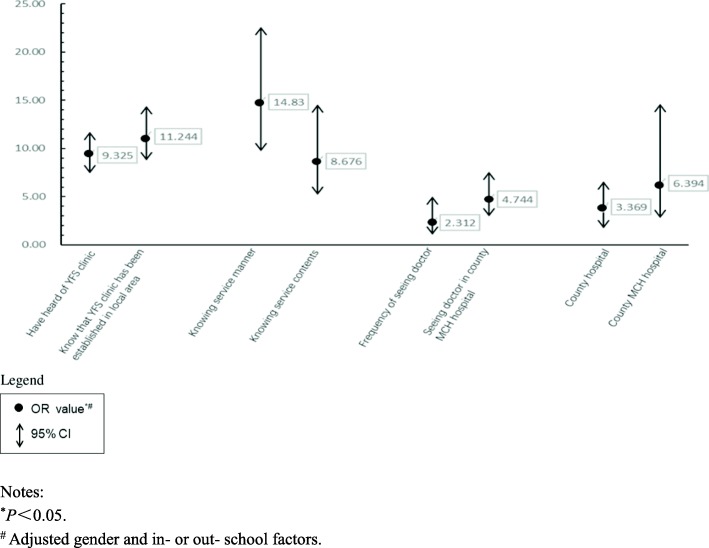
Binary Logistic Regression Analysis on the Effect of Intervention. Notes: ^*^P<0.05. ^#^ Adjusted gender and in- or out- school factors

Knowing at least 4 of 5 characters including site, target population, time and price, hotline number of YFS clinic was seen as knowing service manner. Knowing at least 9 of 12 content variables was seen as knowing service contents. The Binary logistic regression analysis on intervention-control grouping and pre-post intervention showed that the increment of knowledge and satisfaction degrees on the services have statistically significantly closely relationships with the intervention (*P* < 0.05) (Fig. 7).

## Discussion

The study which is an ecology-like one showed that the multi- sectoral cooperation intervention had improved both knowledge and satisfactory degrees on YFS effectively in young people. The increasing of knowledge degree on YFS manner (*P* < 0.05, *OR* = 14.830, 95% CI: 9.728~22.607) and local YFS clinic (*P* < 0.05, *OR* = 11.244, 95% CI: 8.780~14.399) had the closest relationship with the intervention (*P* < 0.05). In another paper about this study, the service quality in interventional areas increased considerably for more than twice of that before intervention while only no more than once in control areas in total [18]. This dominates that the utilization on the service has been improved finally. Maybe the deepened understanding on YFS was helpful for YFS’ multiple characters to inosculate multiple needs of young people in RH, thus it is a beneficial basis to generate good interaction between young people and the services which increase the opportunity to utilize the service by young people. However, in control areas that had not received systematic intervention, even the rates about “Knowing that service price is lower than that of adults”, “Knowing the service includes counseling on physical development, psychological problems and nutrition” etc. were better than those interventional areas before intervention period, they became worse after the intervention period which was showed in all groups of young people and more obvious in in-school group. So, the multi-sectoral intervention in a planned way just after the establishment of YFS to improve the awareness and utilization on YFS may produce a positive effect.

Several studies showed that outreach activities and social mobilization that put health education as priority were quite effective to promote the utilization of YFS or even more effective than service delivery itself, although some researches did not show statistically significant differences or set up control [19–22]. While, by an intervention with control, we established a structured multi-sectoral mechanism to deliver not only services but also health education on YFS in young people as well as create supportive public environment, and obtained a positive output. The education on YFS is also a critical way to provide RH information and education.

Some study said that service provider’s attitude on YFS affected its utilization: positive attitude is beneficial to the utilization, while negative attitude is the barrier and even slower the reducing of sexual transmitted infection and un-wanted pregnancy [23]. The strengthened relationship on YRH between service providers and other sectors through multi-sectoral cooperation mechanism including young people’s participation might improve the attitudes of service providers on YFS which also did good to the utilization. So, the construction of a special multi-sectoral cooperation mechanism to promote the delivery and utilization of YRH service can be considered as an important component of YFS. It is suggested to master the construction skills by YFS providers during the planning of YFS, and the skills should be strengthened continuously through capacity building. Service providers need to be skillful in not only providing correct information to young people through both effective interpersonal communications and regular population education, but also advocating government, identifying and organizing multi-sectors, clarifying responsibilities and strengthening cooperation to create a good social environment for YFS utilization.

In this study, the knowledge rates in out-school young people of two control areas increased statistically significantly. Some studies showed that out-school young people had more risk behaviors in RH than in-school ones so that they had stronger needs for RH service [24, 25]. So, out-school young people paid more attention on YFS perhaps. The detailed reasons need to be explored by further investigation. Whether the increasing of their knowledge degree on YFS through effective intervention be helpful to promote their knowledge degree on pre-marriage and maternity health care services can be proved by further follow-up investigation.

## Conclusions

Social network theory is a helpful tool to facilitate the construction of an effective multi-sectorial cooperation mechanism to promote the awareness and satisfactory degree of YRH services. The results above are important references to guide establishment of multi-sector cooperation mechanisms in the field of young people reproductive health promotion or even public health to coordinate each sector’s role in the cooperation framework aiming to intervention objectives as well as overcome related obstacles and weaknesses in multi-sectoral cooperation.

## Data Availability

All data generated or analyzed during this study are included in this published article.

## Abbreviations

CYN: China Youth Network
GDPs: Gross Domestic Products
MCH: Maternal and Child Health
RH: Reproductive health
SNT: Social network theory
YFS: Youth friendly services
YRH: Young people reproductive health
